# THE SAGE SYMPOSIUM: A MODEL FOR HANDS-ON INTERACTIVE CONTINUING EDUCATION

**DOI:** 10.1093/geroni/igz038.2658

**Published:** 2019-11-08

**Authors:** Becky Powers

**Affiliations:** South Texas Veterans Health Care System Geriatric Research Education and Clinical Center, San Antonio, Texas, United States

## Abstract

Current graduate and postgraduate medical education has minimal geriatric and palliative care curricular requirements, leaving Continuing Education (CE) programs poised to fill a critical educational niche. The San Antonio Geriatric and Palliative Education (SAGE) Symposium was a 3 day long interprofessional CE conference for providers caring for older adults. SAGE addressed geriatric knowledge, skill, and attitude deficits in practicing providers by incorporating: 1) a community and provider based needs assessment, 2) active skills sessions culminating in a health fair, and 3) multimedia based reflective exercises. Needs Assessment (Knowledge): A video needs assessments of older adults were performed using a convenience sampling methodology in 13 non-healthcare public locations in each quadrant of the city. 23 respondents were interviewed before reaching thematic saturation with 3 main themes: geriatric syndromes, patient-provider relationships, and support. Content areas for the course were derived from the needs assessment. Skills Sessions (Skills): In addition to standard plenary sessions, multiple active breakout session taught attendees common geriatric skills. On the last day of the conference, attendees applied these skills under supervision at a senior community health fair. Reflective Exercises (Attitudes): Videos, poems, and artwork with themes on aging were displayed during conference breaks. Attendees received CE credit for electronically submitting short reflections to each multimedia piece. Reflections were compiled and reported back to the group at the end of the conference. By changing the traditional CE conference format to an interactive experience, the SAGE Symposium was able to address knowledge, skills, and attitudes towards aging in its attendees.

